# Comparison of Epson scanner quality for radiochromic film evaluation

**DOI:** 10.1120/jacmp.v13i5.3957

**Published:** 2012-09-06

**Authors:** Hani Alnawaf, Peter K.N. Yu, Martin Butson

**Affiliations:** ^1^ South Coast Cancer Network Wollongong Hospital Wollongong NSW Australia; ^2^ Dept. of Physics and Materials Science City University of Hong Kong Kowloon Tong Hong Kong; ^3^ Illawarra Health and Medical Research Institute Centre for Medical Radiation Physics University of Wollongong Gwynneville NSW Australia

**Keywords:** radiochromic film, Gafchromic, dosimetry, scanner uniformity, X‐ray, radiotherapy

## Abstract

Epson Desktop scanners have been quoted as devices which match the characteristics required for the evaluation of radiation dose exposure by radiochromic films. Specifically, models such as the 10000XL have been used successfully for image analysis and are recommended by ISP for dosimetry purposes. This note investigates and compares the scanner characteristics of three Epson desktop scanner models including the Epson 10000XL, V700, and V330. Both of the latter are substantially cheaper models capable of A4 scanning. As the price variation between the V330 and the 10000XL is 20‐fold (based on Australian recommended retail price), cost savings by using the cheaper scanners may be warranted based on results. By a direct comparison of scanner uniformity and reproducibility we can evaluate the accuracy of these scanners for radiochromic film dosimetry. Results have shown that all three scanners can produce adequate scanner uniformity and reproducibility, with the inexpensive V330 producing a standard deviation variation across its landscape direction of 0.7% and 1.2% in the portrait direction (reflection mode). This is compared to the V700 in reflection mode of 0.25% and 0.5% for landscape and portrait directions, respectively, and 0.5% and 0.8% for the 10000XL. In transmission mode, the V700 is comparable in reproducibility to the 10000XL for portrait and landscape mode, whilst the V330 is only capable of scanning in the landscape direction and produces a standard deviation in this direction of 1.0% compared to 0.6% (V700) and 0.25% (10000XL). Results have shown that the V700 and 10000XL are comparable scanners in quality and accuracy with the 10000XL obviously capable of imaging over an A3 area as opposed to an A4 area for the V700. The V330 scanner produced slightly lower accuracy and quality with uncertainties approximately twice as much as the other scanners. However, the results show that the V330 is still an adequate scanner and could be used for radiation dosimetry purposes. As such, if budgetary requirements are limited, the V700 scanner would be the recommended option at a price eight times cheaper than the 10000XL; however, the V330 produces adequate results at a price which is 2.5 times cheaper again. This may be a consideration for smaller institutions or individuals working with radiochromic film dosimetry.

PACS number: 87.55.Qr; 87.56.Fc

## I. INTRODUCTION

Radiochromic film has provided the medical physics community with a new, two‐dimensional dosimeter which can be used for many applications for dosimetry in radiotherapy,^1^, ^4^ and medical imaging.^5^, ^6^ The standard method of film dosimetry is performed using either a desktop scanner^7^, ^9^ or a film digitizer. The desktop scanner method provides the most economical method of scanning, with the scanners ranging in cost from a mere one hundred dollars up to thousands of dollars. Comparatively, this is still inexpensive compared to a film densitometer which can cost tens of thousands of dollars. ISP technology has recommended the Epson 10000XL desktop scanner for EBT2 film dosimetry. This is due to a number of reasons including its relatively uniform response, low UV output, and high level of reproducibility of image results. The Epson software also allows the user to define that no corrections are made to images, thus allowing the exact film image to be analyzed. Epson also has other desktop scanners in its range of products. These include the V700 and the V330 desktop scanners. The V700 is an A4 size scanner capable of both reflection and transmission scanning with similar specifications to the 10000XL scanner in terms of OD range and resolution. The Epson V330 desktop scanner is a much cheaper version of the scanner and is capable of A4 scanning size for reflection mode. It also has the ability to measure transmission film scanning with a width of 3.5 cm along the landscape direction. The current recommended retail price for each scanner at present in Australia is $6265 for the 10000XL, $949 for the V700, and $249 for the V330. This note compares some basic scanner characteristics for these three scanners for EBT2 film dosimetry to assess quality and accuracy of dosimetry. The reasoning for this work is to assess the accuracy versus price for these scanners specifically for centers where the cost of a higher‐priced scanner may preclude the users from purchasing.

## II. MATERIALS AND METHODS

Gafchromic EBT2, radiochromic film (Lot no. A08161004) (expiry date August 2012) has been utilized for the measurement of scanner characteristics for an Epson 10000XL, V700, and V330 desktop scanners. Only one scanner of each model has been tested. Table 1 shows the basic scanner parameters for the three scanners tested.

**Table 1:** Epson scanner parameters.

	*Epson Scanner Parameters*		
*Parameter*	*V330*	*V700*	*10000XL*
Max Resolution (DPI)	12800	12800	12800
Color Resolution	48 bit	48 bit	48 bit
Light Source	white LED	white cold	xenon gas
Cathode Florescent	florescent		
Max OD	3.5	4.0	3.8
Max Size (inches)	11.7×8.3	11.7×8.3	16.6×11.7
Reflection Mode	yes	yes	yes
Trans Mode	yes (landscape only)	yes/A4	yes/A3

The Epson 10000XL is capable of A3 scanning in both reflection and transmission mode, while the V700 can scan in A4 for reflection and transmission. The V330 is capable of scanning in A4 mode for reflection, but can only scan a 3.5 cm wide strip along the landscape direction for transmission mode.

All films were analyzed using the three PC desktop scanner and Image J software (Ver 1.43u; National Institute of Health, Bethesda, MD)^(10)^ on a PC workstation at least 24 hours after irradiation to minimize effects from postirradiation coloration.^(11)^ The films were kept in a light‐proof container when not being analyzed to reduce coloration from ambient light and UV sources.^12^, ^13^ Scans were performed at 50 pixels per inch resolution and analysis was performed using the red channel, per normal Gafchromic film analysis techniques.^14^, ^15^ This meant that pixel density values for analysis where 16 bit (65536) data. The films were examined in both transmission and reflectance modes. When scanning in reflectance mode, 5 sheets of pure white $80\;\text{gm}/m^{2}$ matt paper (Reflex) were placed behind the film to aid in reflection scanning uniformity.^(16)^ These sheets are the common paper used for printing. In reflectance and transmission modes, optical density (OD) for all films was calculated to evaluate uniformity response in landscape and portrait directions. OD is defined as:

(1) $$\text{OD}=\log\;(65536/P_{t})$$

where $P_{t}$ is the pixel value of intensity through the EBT2 film. All scanner properties were kept the same for transmission scanning, except that the light source used was the scanners transmission light source (as opposed to the reflection method light source) and the white backing was removed. The films when scanned were always positioned in the same manner to eliminate differences in results caused by film polarization effects.^17^, ^18^ The films were placed in the same position on each scanner to eliminate variations in response caused by the film's nonuniformity. For data analysis, the outer 2 cm edge of the scanned film results was removed. This was performed to minimize any effects on scanner results from film edges or cutting damage.^(19)^ Results given are the average for five scans of each film piece with a 1 cm wide profile in either the landscape or portrait direction. The 1 cm wide profile is an average of the pixel values obtained within this region (X direction) with the profile performed in the Y direction. Experiments were repeated 5 times for analysis using different films, with results shown as the average of five scans for one film piece. No substantial variation in uncertainty or results was seen over the five experiments performed.

## III. RESULTS & DISCUSSION

(Figure 1a) shows a profile scan of a nonirradiated EBT2 film in the portrait direction of the scanner (and film) when scanned in reflection mode on the Epson 10000XL, V700, and V330 scanners. Results are normalized to 1 as the average OD reading for each individual film analyzed. This removes the variations in OD measured between the different scanners caused by the differing lights sources of each scanner type. As can be seen, each scanner produces a different output, with the variations seen in the portrait direction appearing to be scanner‐specific rather than any film‐based variations in this instance. The standard deviations in output for the three scanners across this profile were 0.6%, 0.4%, and 1.0% for the 10000XL, V700, V330, respectively. The minimum/maximum variations were approximately 2.5% — 2% for the 10000XL and V700, and 3.5% for the V330.

**Figure 1(a):**
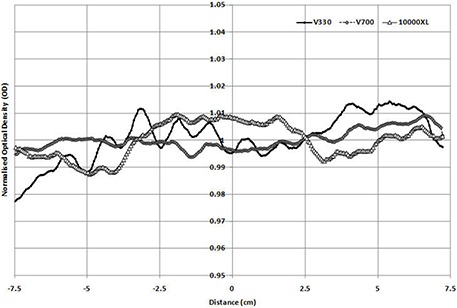
Normalized profile in portrait direction (nonirradiated film).

(Figure 1b) shows similar results for the same film and scanners, but in the landscape direction. Here again, the standard deviation in output were found to be 0.5%, 0.3%, and 0.7% for the 10000XL, V700, and V330, respectively. The maximum/minimum values were approximately 2% (10000XL and V700) and 3% (V330).

When an irradiated film was scanned (e.g., 3 Gy), (Figs. 2a)and (2b) are produced for the portrait and landscape directions, respectively. Standard deviations of normalized results across the film were calculated to be 0.85%, 0.5%, and 1.16% for the 10000XL, V700, and V330, respectively, in portrait mode, and 0.2%, 0.2%, and 0.44% for landscape direction. These results showed that in reflection mode scanning, the 10000XL and the V700 were superior (and similar) in reproducibility and uniformity. The V330 produced larger nonuniformity in scanning, however not to the extent which would exclude its use as an adequate desktop scanner for radiochromic film analysis.

**Figure 1(b):**
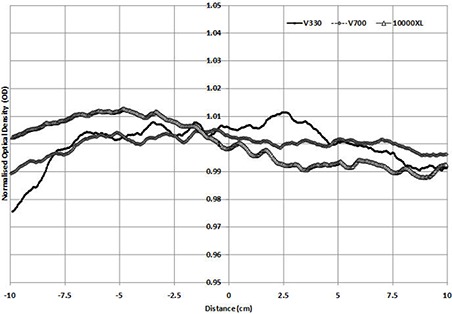
Normalized profile in landscape direction (nonirradiated film).

When scanning is performed in transmission mode, (Figs. 3a)and (3b) are produced for portrait and landscape modes. As the V330 was unable to scan transmission in the portrait direction, only 10000XL and V700 results are shown. Both profiles show the well‐known portrait direction nonuniformity response (transmission mode), which is similar in magnitude for both scanners with a standard deviation across the portrait direction of 1.71% and 1.78%, respectively for the 10000XL and V700. When scanning is performed in the landscape direction, the 10000XL and the V700 have standard deviations of 0.25% and 0.6%, and the V330 produces a 1% standard deviation variation. The figure shows noticeable bumps in scan results for the V330 scan, with up to 4% maximum/minimum values. These variations are of the order of 1.5% for the 10000XL and 2% for the V700.

**Figure 2(a):**
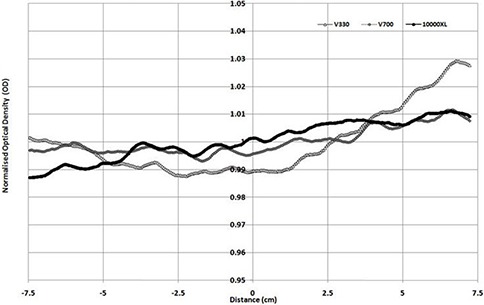
Normalized profile in portrait direction — 3 Gy irradiation.

**Figure 2(b):**
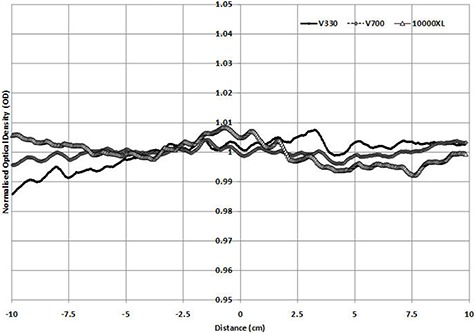
Normalized profile in landscape direction — 3 Gy irradiation.

Only one scanner of each type (V330, V700, 10000XL) was tested, so we cannot eliminate variations in base scanner quality in this work. However, each scanner was purchased new and no physical or software defects were seen during the testing procedures.

In comparison, both the 10000XL and the V700 scanners provide relatively similar and high‐quality scanning analysis systems, which are both accurate and provide low levels of nonuniformity in reflection mode. Both have a higher level of scanner nonuniformity in transmission mode, portrait direction. The V330 also provides adequate scanning accuracy and low nonuniformity in reflection mode, albeit slightly larger than the other two scanners. It does, however, provide an adequate level of accuracy in this mode for dosimetry purposes. Since the scanner nonuniformity in transmission mode was larger, combined with the fact that scanning could only be performed in the landscape direction, this method of scanning with the V330 is not recommended. In terms of dosimetric changes, results have shown that the V330 in reflection mode produces a maximum standard deviation across profile measurements of 1.2%, with maximum/minimum variations of 3.5%. For a dose level of 200 cGy, this equates to average variations of 2.4 cGy and max/min dose error of 7 cGy. This is compared to the V700 with values of (1 cGy, 5 cGy) and the 10000XL (1.6 cGy, 4 cGy). Obviously in some radiotherapy or research centers, the cost of the Epson 10000XL could be a limiting factor for purchase and, as such, we could recommend both the V700 and the V330 for dosimetry, with the V700 being the next obvious choice for accuracy.

**Figure 3(a):**
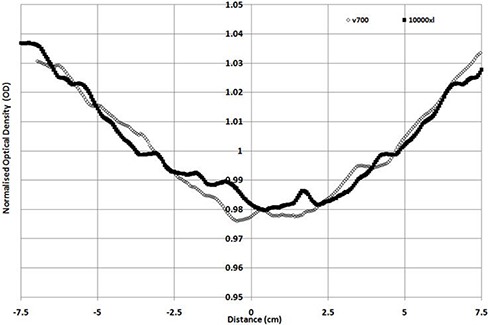
Normalized profile in transmission mode (portrait direction).

**Figure 3(b):**
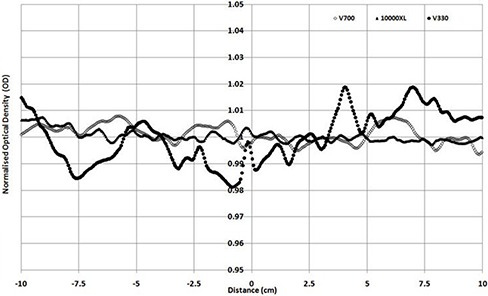
Normalized profile in transmission mode (landscape direction).

## IV. CONCLUSIONS

This work has compared three types of Epson flatbed scanners to assess the properties for accurate radiation dosimetry with EBT2 Gafchromic film. Results have shown that either the Epson 10000XL or the Epson V700 provide similar accuracy and reproducibility, and would be the recommended scanners for use. The V700 provides a significant cost saving over the 10000XL and produces the same level of accuracy, albeit with only an A4 scanning size. The V330, which is a much cheaper entry level scanner, did not provide the same level of accuracy as the other Epson scanners; however, results showed that an adequate level of accuracy and reproducibility was available in reflection mode scanning. Its low cost would make it suitable for individuals or centers with limited funds available to purchase such equipment. The V700 would be the recommended scanner if cost‐to‐accuracy ratios were considered.

## ACKNOWLEDGMENTS

This work has been fully supported by a grant from the Research Grants Council of HK‐SAR, China (Project No. CityU 123810). Hani Alnawaf was supported by the Saudi Arabian Government.
